# Stress hyper-reactivity increases vulnerability to developing binge-type eating and associated anxiety-like behavior; comparison between Wistar-Kyoto and Sprague-Dawley rats

**DOI:** 10.3389/fnut.2024.1368111

**Published:** 2024-04-04

**Authors:** Daniela Sarai Rodríguez-Rangel, Erika Estrada-Camarena, Carolina López-Rubalcava

**Affiliations:** ^1^Departamento de Farmacobiología, Centro de Investigación y Estudios Avanzados (CINVESTAV-Sede Sur), Mexico City, Mexico; ^2^Laboratorio de Neuropsicofarmacología, Dirección de Neurociencias, Instituto Nacional de Psiquiatría Ramón de la Fuente Muñiz, Mexico City, Mexico

**Keywords:** binge eating, anxiety, Wistar Kyoto rats, stress hyper-reactivity, corticosterone

## Abstract

**Introduction:**

Binge eating disorder (BED) is a widespread eating disorder that primarily affects women worldwide, and it is characterized by the presence of binge eating episodes and the absence of any compensatory behavior to prevent weight gain. BED presents elevated comorbidity with other psychiatric disorders, such as anxiety, and it has been suggested that stress sensibility could be a vulnerability factor for the development of BED and the associated anxiety comorbidity. In this study, we aim to investigate whether the Wistar-Kyoto rat strain (WKY), which has a stress hyper-reactive phenotype, could develop both binge-type eating and anxiety-like behaviors simultaneously. We also aim to compare its vulnerability to developing both behaviors with the Sprague Dawley rat strain (SD), a rat strain commonly used in binge-eating models.

**Methods:**

WKY and SD rats were subjected to the model of intermittent access to palatable food (sucrose solution 30% or shortening) without calorie restriction or stress exposure. We evaluated and compared the development of binge-type eating behavior, anxiety-like behavior, and serum corticosterone variation as an index of the stress response in both rat strains.

**Results:**

WKY rats presented a higher percentage of binge-type eaters and required less time to develop binge-type eating behavior than SD rats. The WKY eating pattern emulated a binge-eating episode regardless of the palatable food. Although the development of sucrose binge-type eating was similar between strains, WKY developed more easily the shortening binge-type eating than SD and was more susceptible to developing anxiety-like behavior. Additionally, sucrose binge eating seems to differentially affect both strains’ hypothalamic-pituitary-adrenal (HPA) axis response to stress since it facilitated its response in SD and blunted it in WKY.

**Discussion:**

Our results show that high-stress sensitive phenotype is a common vulnerability factor for the development of binge-type eating and anxiety-like behavior. Regardless of the macronutrient composition of the palatable food, WKY is susceptible to developing a binge-type eating behavior and is more susceptible than SD to developing anxiety-like behavior simultaneously. In conclusion, results showed that a hyper-reactive stress phenotype predisposes the development of binge-type eating behavior and anxiety-like behavior in the absence of calorie restriction and stress exposure.

## 1 Introduction

Binge eating disorder (BED) is the most common eating disorder worldwide (1), with an estimated global prevalence of 0.9%. It is more common in women than men, as is the case with other eating disorders (2), and the age of onset is around late adolescence and emerging adulthood (3, 4). BED is characterized by the presence of binge eating episodes, defined as the consumption of large amounts of food in the absence of hunger in a short time-lapse, without presenting any compensatory behavior (5). During these binge eating episodes, patients consume mainly palatable food high in fat and/or carbohydrates and experience a “loss of control” over their eating behavior and severe psychological distress (5).

BED presents elevated comorbidity with other psychiatric disorders, such as anxiety. Patients with psychiatric comorbidity usually present a more severe disorder than patients without comorbidity; this is reflected in an earlier age of development, more frequent binges, and fewer results in their treatment (6). It has been estimated that nearly 59% of BED patients suffer from anxiety disorders (7), and some clinical studies have suggested that anxiety disorder could be a predisposition factor for the development of BED (8, 9). One proposed explanation of the high comorbidity is that anxiety disorder and eating disorders may be related by sharing common vulnerability factors (10, 11), and one possible factor is a high-stress reactivity (12), defined as an exaggerated behavioral and neuroendocrine response to a stressor that does not align with the stressor’s threat (12, 13). In this way, binge eating could emerge as a maladaptive coping behavior to stress (8, 9). At the same time, the hyperreactivity of the hypothalamic–pituitary–adrenal (HPA) axis could lead to the development of an anxiety disorder (13), resulting in the simultaneous development of both disorders.

Several animal models have been developed to resemble the etiology of BED to understand the metabolic and neural alterations associated with it. The most frequently used model is female Sprague Dawley rats (SD) (14); however, although SD can successfully develop a binge-type eating behavior using sucrose, fat, or more complex palatable foods, it does not express any anxiety-like behavior (15, 16), even when a stressful stimulus is employed to induce the binge eating (17). Therefore, it is relevant to explore if a high-stress sensitive strain develops binge eating and anxiety simultaneously.

In a previous study, our group demonstrated that female Wistar-Kyoto (WKY) rats developed sugar-binging and associated anxiety-like behavior in an intermittent access model without calorie restriction (18). Since the WKY strain is characterized by expressing a hyper-reactive HPA axis (19, 20) and has a high predisposition to developed anxiety and depressive-like behaviors (21, 22), we proposed the WKY rat strain as an animal model to evaluate whether stress-vulnerability contributes to developing a binge-eating-anxiety comorbidity state.

Considering the idea of high-stress reactivity as a common vulnerability factor in the development of BED and anxiety disorders, we hypothesize that the WKY’s stressful phenotype might play as a common vulnerability factor in the development of binge-type eating and associated anxiety-like behaviors, regardless of the palatable food employed. We believe this predisposition could make the WKY rat strain more susceptible to developing these behaviors than other strains.

To test this hypothesis, we subjected female WKY and SD rats in their emerging adulthood to an intermittent palatable food model using sucrose or shortening as palatable foods. We compare the development of the binge-type eating behavior in both strains of rats by analyzing (a) the binge-type eating intake, (b) the susceptibility to develop abnormal eating behavior, and (c) the duration of the binge-eating episodes. Once the binge-type eating behavior was established, we evaluated the effect of the abnormal eating pattern on the anxiety-like behavior in two animal models of anxiety. Also, the serum corticosterone stress response was analyzed as an index of HPA axis activation.

## 2 Methods

### 2.1 Animals

Because the age of onset of BED is around late adolescence and emerging adulthood, we used 7-week-old (adolescent) female Sprague Dawley (SD) and Wistar Kyoto (WKY) rats provided by our breeding facilities. Rats were group-housed (5 per cage) in wire-topped, acrylic cages (43x53x21cm) and maintained in an inverted light schedule (12 h/12 h light/dark cycle, lights on 22:00 h) with a controlled environment (22 ± 2°C, 50 ± 10% humidity) and free access to water and standard food (LabDiet 5,008, PMI Nutrition International, LLC).

All experimental procedures were approved by CINVESTAV’s ethics committee (CICUAL) (Protocol 0179–16) and followed the regulations established by the Mexican Official Norm (NOM-062-ZOO-1999) for the use and care of laboratory animals.

### 2.2 Binge-type eating induction protocol

We use the same binge-type eating induction protocol as in previous work (18). This protocol is based on intermittent palatable food access (23). Briefly, after one habituation week, rats were subjected to three training sessions, one session per day, to avoid handling and separation-related stress during subsequent tests. The training sessions consisted of weighing and isolating the rats in individual cages for 2 h with chow-standard food and water *ad libitum*. Once the time was up, the rats were returned to their home cage.

After the last isolation session, three groups for each strain were randomly formed based on the food used to induce the binge-type eating behavior: (1) control: standard food; (2) Sucrose: standard food +30% sucrose solution; (3) Shortening: standard food + vegetable shortening (Crisco® All-Vegetable shortening, J.M Smucker Co., Orrville, OH).

To avoid neophobia development in the sucrose and shortening groups, we provided their respective palatable food *ad libitum* in their home cage for 24 h along with their regular food. Following the presentation of the palatable food, the rats were left untouched for 48 h before initiating the intermittent palatable food access sessions. By this time, rats were approximately 9 weeks old, equivalent to their emerging adulthood (24).

**Figure 1:**
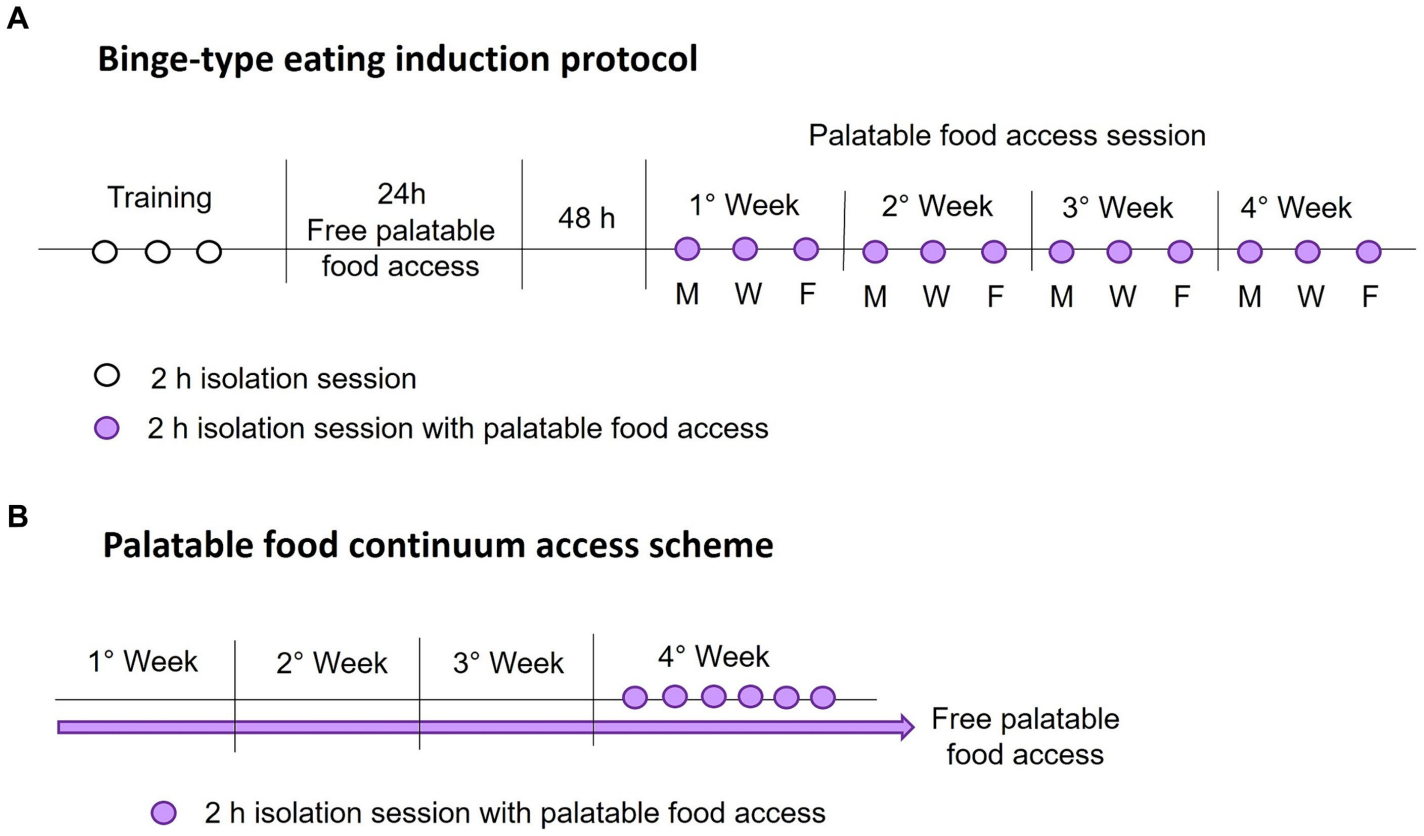
Schematic representations of the binge-type eating induction protocol **(A)**, with intermittent palatable food access on Monday (M), Wednesday (W), and Friday (F), and the palatable food continuum access scheme **(B)**.

The intermittent palatable food access without calorie restriction consisted of allowing access to palatable food for a 2-hour session every 2 days on Monday, Wednesday, and Friday each week. During each food access session, rats were weighed and placed in individual cages with free access to water and a known amount of standard and palatable food (only the sucrose and shortening groups). Once the time was up, the rats were returned to their home cage with standard food *ad libitum*. All isolation sessions were performed 3 hours after the light went out. After each session, we weighed the food and calculated the calorie intake. Figure 1A shows a schematic representation of the binge-type eating induction protocol.

### 2.3 Classification criteria

We identified the animal that expressed a binge-type eating behavior by comparing its consumption against animals with continuum access to palatable food. For this purpose, we implemented two groups per strain (*n* = 6) with *ad libitum* access to chow and one of the palatable foods, sucrose solution (30%) or vegetable shortening, for 4 weeks. We measured the calorie consumption and animal weight daily to ensure constant consumption. In the last week, rats underwent six isolation sessions of 2 h, one daily, with access to a known amount of standard and palatable food. We evaluated the calorie consumption during the isolation session similarly to the binge-type eating induction protocol. Figure 1B shows a schematic representation of the implemented palatable food continuum access scheme.

Using a student’s *t*-test, we compare the kilocalorie consumption during the isolation sessions of the groups with continuum palatable food access against the binge-type eating induction protocol groups. We consider that an animal expressed a binge-type eating behavior if the kilocalorie consumption of its last six isolation sessions was significantly higher than the average consumption in isolation of the animals with continuous access to the respective palatable food.

### 2.4 Anxiety-like behavior evaluation

#### 2.4.1 Elevated plus maze

The elevated plus maze (EPM) test analyzed anxiety-like behavior. We performed the test according to Walf & Frye (25). Briefly, under red light, the animal was placed in the center of a cross-shaped raised arena with two open arms (without walls) and two closed arms (with walls); the animal was allowed to roam for 5 min. We quantified the entrances to open and closed arms and the time spent in open arms. Since the elevated plus maze is a conflict test that counterposes the rat’s natural curiosity to explore new areas against its natural aversion to high and open spaces, a low permanence and few entries to open arms are interpreted as increased anxiety-like behavior. Results are shown as the percentage of entries to open arms, calculated concerning the total entries (open + closed arms) and the percentage of the total time spent in open arms.

#### 2.4.2 Modified marble burying test

We performed a test similar to the marble burying test described by Thomas et al. (26). We implemented the same experimental conditions, but instead of considering the number of buried marbles as a measure of the anxiety-like behavior, we measured the time spent by the rat in burying and compulsive rostral grooming. The change in the evaluation criteria was due to the lack of rats’ aversion toward the marbles and the observation of a burying behavior aimed at seeking an escape route instead of burying the marbles. Since the compulsive rostral-grooming and burying behaviors have been described as active coping strategies to decrease the impact of stress and associated anxiety (27, 28), a longer time performing these behaviors can be interpreted as an increase in anxiety-like behavior.

Briefly, rats were individually placed in a transparent acrylic cage (43×53×21cm) with fresh chip wood bedding (5 cm deep) and 20 glass marbles (1.5 cm diameter) in a 4×5 arrangement on top. The test was performed under red light and videotaped for 5 min. We quantified each animal’s cumulative time burying and performing compulsive rostral grooming.

### 2.5 Experimental procedure

#### 2.5.1 Evaluation of each rat strain’s palatable food consumption and binge-type eating susceptibility

We analyzed the animal weight and the kilocalorie consumption throughout the 12 palatable food access sessions of a pull of experimental batches of WKY and SD rats (Control: SD *n* = 43, WKY *n* = 37; Sucrose: SD *n* = 49, WKY *n* = 43; Shortening: SD *n* = 52, WKY *n* = 42) subjected to the binge-type eating induction protocol.

First, we evaluated the average kilocalorie consumption per rat of sucrose and shortening groups of both strains. Subsequently, we applied the classification criteria described in section 2.3 to identify the animals that developed sugar or shortening binge-type eating behavior.

Once we identified the binge-type eaters, we evaluated the time these animals required to develop the binge-type eating behavior. For this purpose, we re-applied the classification criteria, but this time, we compared the palatable food consumption of their first six isolation sessions of the binge induction protocol. In this way, we identified the animals that developed binge-type eating behavior within only 2 weeks of the intermittent palatable food access sessions.

We evaluate the strain susceptibility to develop the binge-type eating behavior by comparing between strains, the proportion of rats classified as binge-eaters, and the proportion of binge-eaters that required only 2 weeks to express the abnormal eating behavior.

#### 2.5.2 Characterization of the palatable food eating pattern during an isolation session (duration of the binge-eating episode)

We analyzed the effective time each rat employed to consume the palatable food during an isolation session to evaluate if the consumption pattern resembled the main characteristic of a binge episode: consuming a large amount of food in a short time.

Thus, we videotaped the last isolation session of 9 sucrose and 7 shortening binge-type eaters of each strain. At the end of the session, we quantified the palatable food calorie consumption. In the video, we quantified the cumulative time each animal spent consuming the palatable food and its distribution across the 2-h session divided into 15-min lapses expressed as a percentage of the total consumption time.

#### 2.5.3 Evaluation of anxiety-like behavior

The evaluation of anxiety-like behavior was performed only in animals classified as binge-eaters. We used the elevated plus maze and the modified marble burying tests to assess the anxiety-like behavior associated with binge-type eating behavior. Both tests were performed 24 h after the last isolation session.

The EPM test was applied in an independent group of rats divided into control (SD and WKY *n* = 14), sucrose (SD *n* = 9, WKY *n* = 10), and shortening (SD and WKY *n* = 14) groups exposed to the binge-type eating induction protocol.

The modified marble burying test was applied to independent groups of rats, divided into control (SD *n* = 10, WKY *n* = 9), sucrose (SD *n* = 7, WKY *n* = 10), and shortening groups (SD *n* = 10, WKY *n* = 9), exposed to the binge-type eating induction protocol.

#### 2.5.4 Determination of the corticosterone response to a stressor

As an index of the HPA axis activation, we evaluated the serum corticosterone in non-stressful and stressful conditions. For the stressful conditions, animals classified as binge-type eaters were individually placed, for 10 min, in an acrylic cage (16×18×29) with an electrified prod located in one of the cage walls that produced a discharge of 0.3 mA to the touch (LaFayette Instruments Co., model 5,806, Lafayette, Indiana, USA).

We used a batch of 10 animals per group, per strain, subjected to the binge induction protocol. After identifying the binge-type eaters of the sucrose and shortening groups, all groups were divided into two equal parts. Half of each group was euthanized by decapitation 1 day after the last palatable food access session, and the blood trunk was collected. The remaining group experienced an additional palatable food access session, and 24 h later, the group was subjected to stress conditions. Animals were euthanized immediately after, and the blood trunk was collected. All animals were euthanized 3 hours after the light went out.

The blood samples were centrifuged (3,000 g, 20 min, 4°C), and serum samples were collected and stored at −80°C until corticosterone determination was performed using an enzyme-linked immunosorbent assay (ELISA) kit (Enzo, ADI-9009097), according to manufacturer instructions.

### 2.6 Data analysis

All statistical analyses were performed using GraphPad Prism 8.0.1 software. Before any statistical analysis, a normality test was applied using the Shapiro–Wilk test to determine whether sample data had been drawn from a normally distributed population.

The Student’s *t*-test was used to compare between strains the average caloric consumption of each “Diet” during the induction protocol and for the classification of binge-type eaters by comparing intermittent access vs continuous access.

The Fisher test was used to compare between strains the different proportions of animals classified as binge-type eaters and the proportion of binge-type eaters that developed the abnormal behavior within 2 weeks of palatable food access sessions.

A Repeated measure two-way ANOVA was used to assess the body weight variation during the binge-type eating induction protocol, considering isolation session and strain as factors.

Two-way ANOVA tests were used for the following analysis: (a) Effect of diet on anxiety-like behavior, taking strain and diet as factors; (b) For the analysis of the time distribution of palatable food consumption across one access session, taking time and strain as factors; (c) To compare the corticosterone stress response of each strain for each diet condition, taking strain and stress condition as factors.

## 3 Results

### 3.1 Induction and evaluation of the binge-type eating susceptibility of each rat strain

#### 3.1.1 Body weight variation across the binge-type eating induction protocol

**Figure 2:**
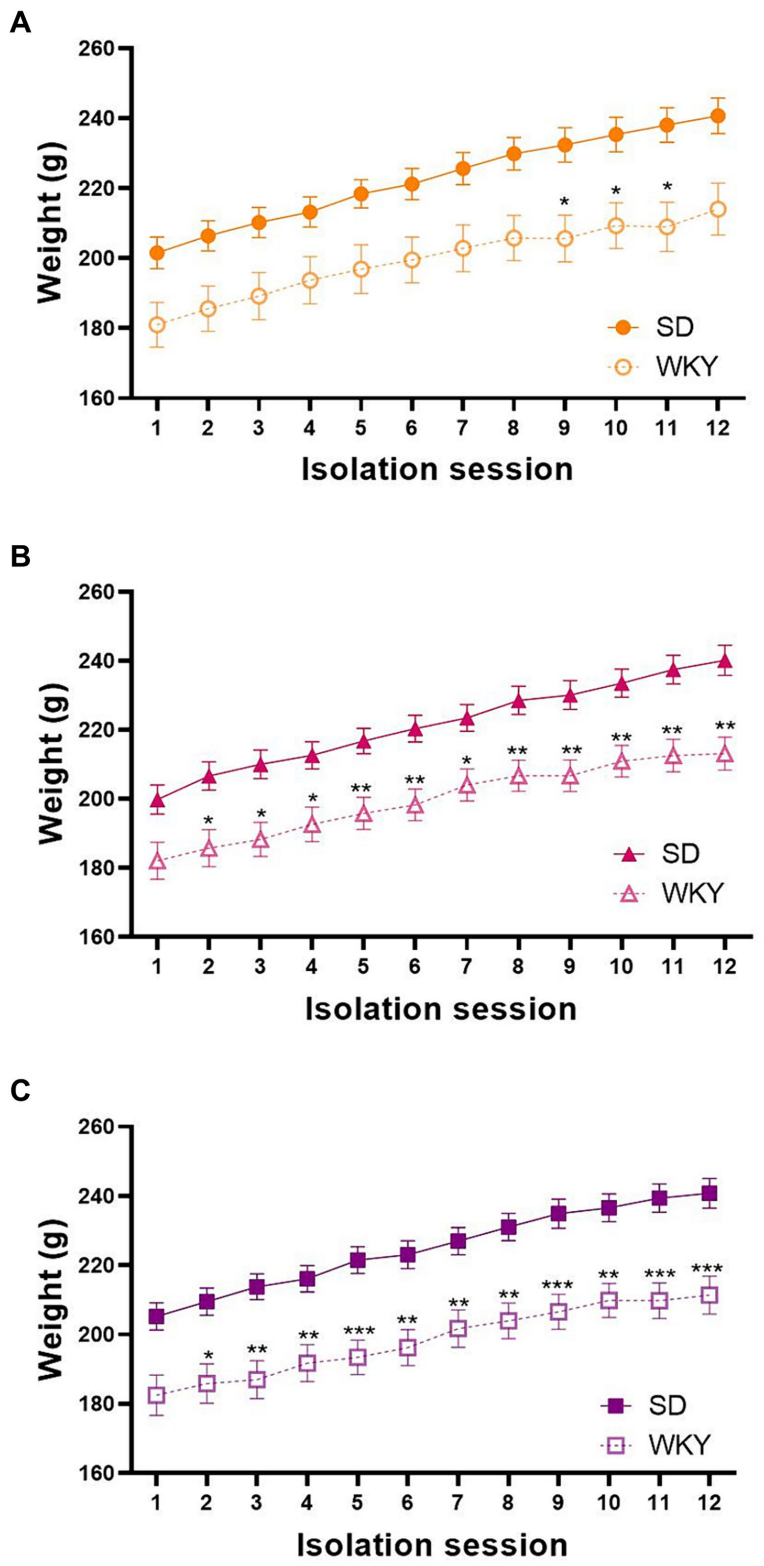
Body weight of control **(A)**, sucrose **(B)**, and shortening **(C)** groups across the 12 isolation sessions of the binge-type eating induction protocol. Mean ± SEM. RM two-way ANOVA (analysis available on Supplementary material). *Post-hoc*: Sidak’s, * vs. SD *p < 0.05, ***p < 0.001. Control: SD *n* = 39, WKY *n* = 37; Sucrose: SD *n* = 49, WKY *n* = 43; Shortening: SD *n* = 52, WKY *n* = 42.

As we employed two different rat strains, we measured their body weight before each isolation session to identify differences in their weight gain across the binge-type induction protocol. We performed a repeated measure two-way ANOVA for each group, using the strain and the isolation session as variation factors. In general, the strain, the isolation session, and the interaction of both factors were sources of variation in the three experimental groups. Even though both strains similarly gain weight across the experimental protocol, Sidak’s multiple comparisons showed a significantly lower weight of WKY in comparison with SD starting from session 4 in control groups, session 6 in sucrose groups, and session 3 in shortening groups (Figure 2).

#### 3.1.2 Analysis of the palatable food consumption dispersion for each rat strain

Before applying the classification criteria, we explore the dispersion of the calorie consumption of control, sucrose, and shortening groups of both strains. Due to the body weight difference between strains, the calorie consumption was normalized concerning the animal’s weight. SD presented higher dispersion than WKY, particularly in the shortening group (Mean ± Std.Dev.; Control: SD-0.05 ± 0.019 vs. WKY-0.05 ± 0.007; Sucrose: SD-0.13 ± 0.023 vs. WKY-0.12 ± 0.017; Shortening: SD-0.21 ± 0.06 vs. WKY-0.26 ± 0.05***).

**Figure 3:**
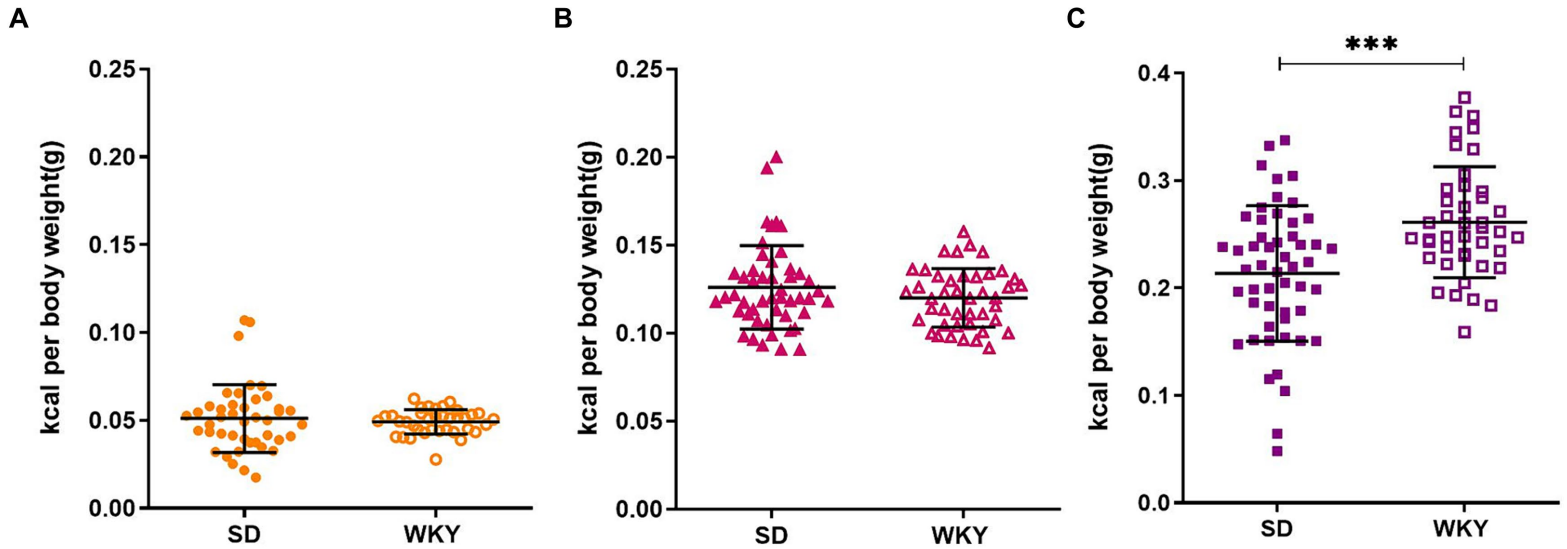
Average caloric intake per rat of the Sprague-Dawley (SD) and Wistar-Kyoto (WKY) strains during the binge-type eating induction protocol of control **(A)** sucrose **(B)** and shortening **(C)** groups. Mean ± SD. *t* student. Control: SD *n* = 39, WKY *n* = 37; Sucrose: SD *n* = 49, WKY *n* = 43; Shortening: SD *n* = 52, WKY *n* = 42, **p < 0.01.

When we compared the average palatable food consumption between strains, we observed that both strains presented a similar chow (control group) and sucrose consumption but differed in the shortening consumption. Specifically, WKY consumed more shortening than SD (*p* = 0.001) (Figure 3).

#### 3.1.3 Determination of the binge-eaters and binge-resistant proportion for each rat strain

**Figure 4:**
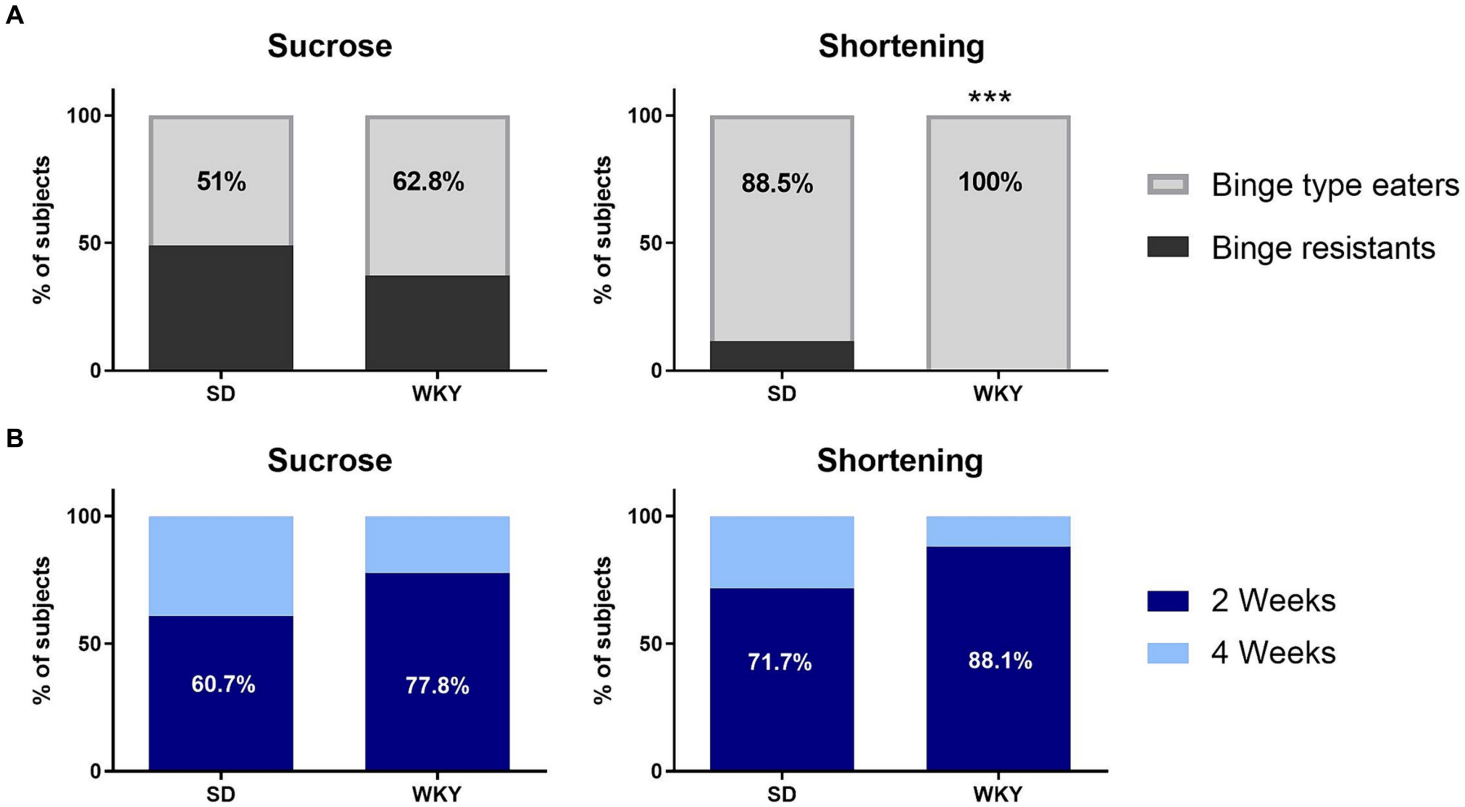
Percentage of Sprague Dawley (SD) and Wistar-Kyoto (WKY) rats resistant and prone to develop binge-type eating behavior when subjected to the binge-type eating induction protocol using sucrose or shortening as palatable food **(A)**; and percentage of sucrose and shortening binge-type eaters of both strains that developed the binge-type eating behavior within only 2 or 4 weeks of the induction protocol **(B)**. Percent. Fisher’s exact test. ***p < 0.0001.

Through a Student’s *t*-test, we identify sucrose and shortening binge-type eaters as those animals with a consumption significantly higher than their counterparts with continuum access. Once we applied the classification criteria, we identified that 51% of the SD and 62.8% of the WKY with intermittent access to sucrose developed a binge-type eating behavior, and in the groups with intermittent access to shortening, 88.5% of SD and 100% of WKY (Figure 4A).

We performed a Fisher’s exact test to compare the proportion of binge-type eaters and binge-resistant animals between strains for each palatable food; as a result, only the shortening groups differ in their proportions since WKY have a higher percent of binge-type eaters than SD (*p* < 0.0001).

#### 3.1.4 Time required to develop the binge-type eating behavior

After identifying the binge-type eaters, we evaluated their required time to develop abnormal eating behavior. Of all the animals identified as sucrose binge-type eaters, 60.7% of the SD and 77.8% of the WKY showed abnormal eating behavior within 2 weeks of the binge-type eating induction protocol. It took the same time for 71.7% of the SD and 88.1% of the WKY shortening binge-type eaters to display abnormal eating behavior (Figure 4B).

We performed a Fisher exact test to compare the proportion of subjects that require 2 or 4 weeks to develop binge-type eating behavior between strains. Even though there was no statistical difference between strains, WKY had 17% more binge-type eaters than SD, which required only 2 weeks of intermittent palatable food access sessions to express the abnormal eating behavior, regardless of the palatable food employed.

### 3.2 Characterization of a binge-type episode during an isolation session

In addition to the high amount of food ingested, another characteristic of a binge eating episode is its short duration. Thus, we considered it relevant to evaluate the effective time that rats spent eating the palatable food, the calorie consumption, and the percent eating time distribution across the 2 h, divided into 15-min lapses of the last access session.

**Figure 5:**
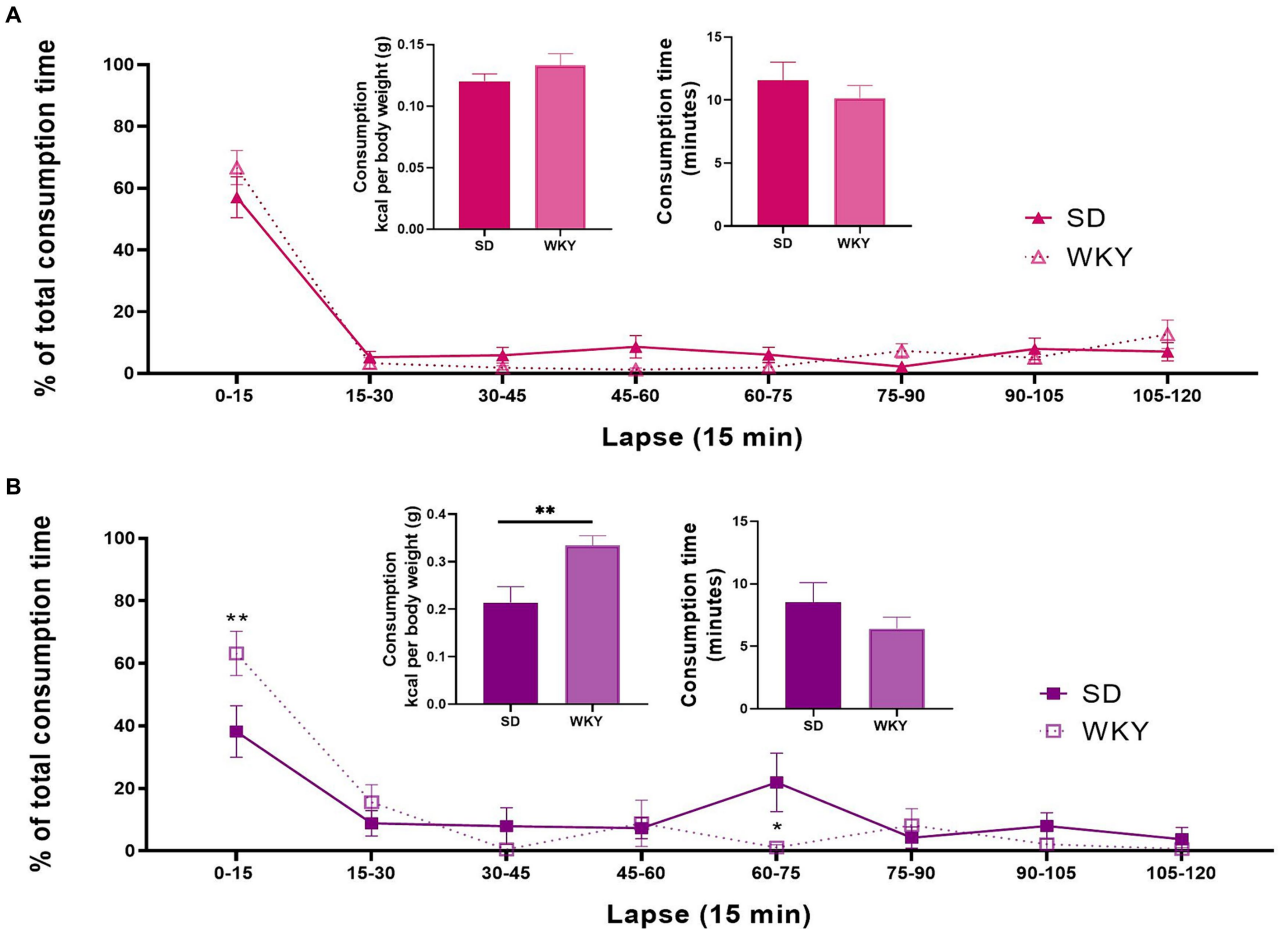
Kilocalorie consumption, total food palatable consumption time, and percent distribution of the consumption time throughout the last 2h-isolation session of the Sprague-Dawley (SD) and Wistar-Kyoto (WKY) rats of the sucrose **(A)** and shortening **(B)** binge-type eaters. Mean ± SEM. Sucrose *n* = 9; Shortening *n* = 7. *t* student for consumption and consumption time. Two-way ANOVA for the percent time distribution, *post hoc*: Sidak’s * vs SD.

We analyzed the kilocalorie and total time consumption using a Student’s *t*-test. We performed a two-way ANOVA to evaluate each palatable food’s total consumption time distribution, using the strain and time-lapse as variation factors. In sucrose binge-type eaters, the calorie consumption, total time consumption, and percent eating time distribution were similar between both strains. The only source of variation in the percent eating time distribution was the time-lapse (*F*_(7, 112)_ = 69.15, *p* < 0.0001) (Figure 5A).

In shortening binge-type eaters, WKY presented a significantly higher kilocalorie consumption than SD (*p* < 0.0093), although the total time consumption was similar between both strains. The sources of variation of the percent time distribution were the time-lapse (*F*_(7, 77)_ = 16.79, *p* < 0.0001) and the interaction of time-lapse and strain (*F*_(7, 77)_ = 2.938, *p* = 0.0088). Sidak’s multiple comparisons between strains showed differences in two time-lapses; in the 0–15 lapse, WKY presented a significantly higher consumption than SD, and in the 60–75 lapse, SD presented a significantly higher consumption than WKY (Figure 5B).

The distribution of the consumption time was similar for the sucrose binge-type eaters of both strains. In both cases, nearly 60% of the total time consumption occurred during the first 15 min of the session. For shortening binge-type eaters, the distribution of the time consumption between strains was different. Thus, in WKY-shortening binge-type eaters, approximately 60% of the consumption time took place during the first 15 min; in contrast, the SD-shortening binge-type eaters presented two stages of consumption, one in the first 15 min and the second on the 60–75 min lapse. In other words, when shortening is used as palatable food in an intermittent access model, WKY emulates a binge-type eating episode better than SD as WKY presented a higher consumption than SD in the same amount of time, and its consumption occurs in only one event.

### 3.3 Evaluation of the anxiety-like behavior

#### 3.3.1 Elevated plus maze test

As a first attempt to evaluate the anxiety-like behavior, we performed the EPM in the control group and rats classified as sucrose and shortening binge-type eaters of both strains.

**Figure 6:**
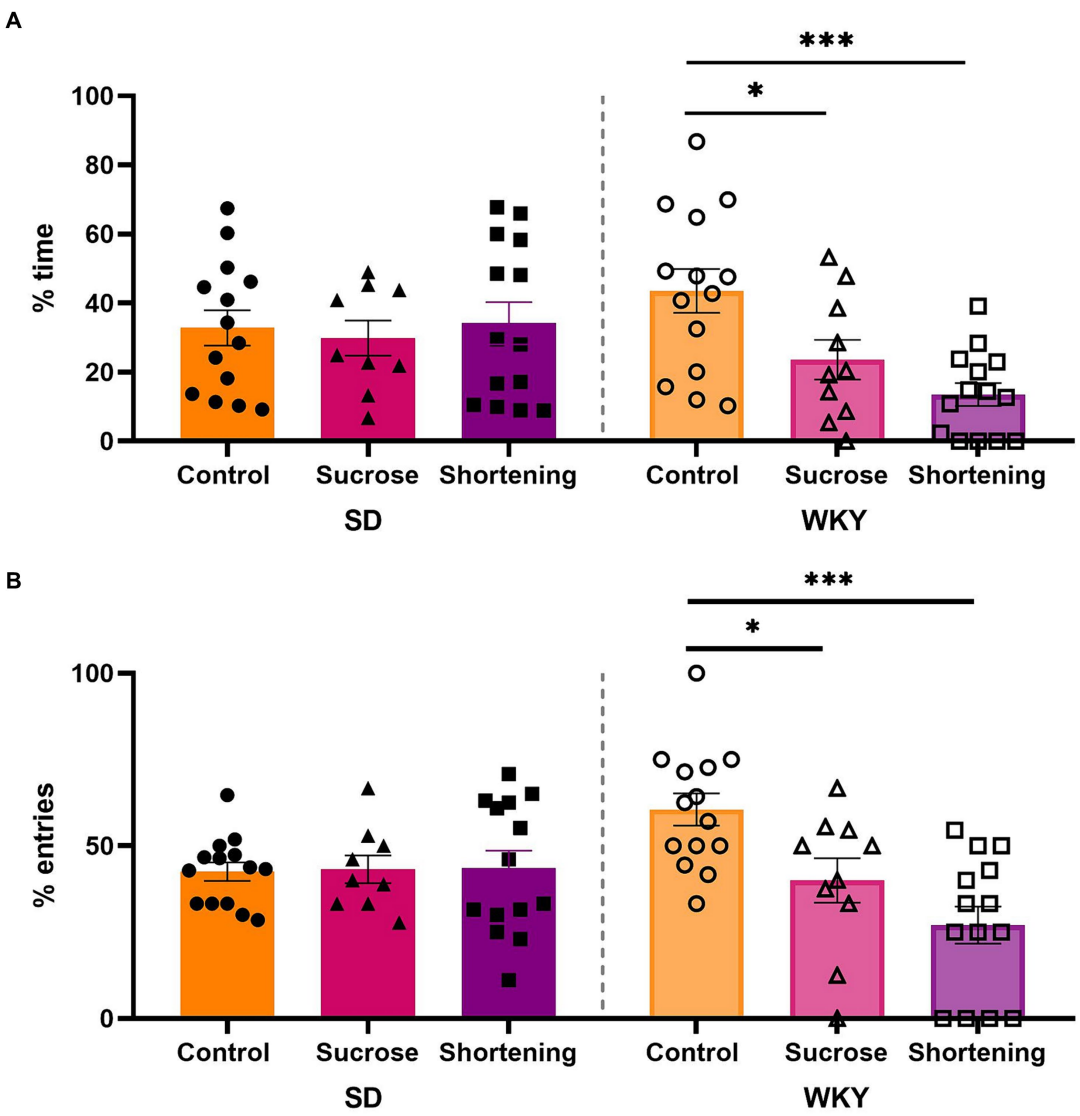
Percentage of time spent in open arms **(A)** and percentage of entries to open arms **(B)** in the elevated plus maze test of control (chow), sucrose, and shortening groups of Sprague-Dawley (SD) and Wistar-Kyoto (WKY) rats subjected to the binge type eating behavior induction protocol. Mean ± SEM. Two-way ANOVA. *Post-hoc*: Tukey, *p < 0.05, ***p < 0.001. *n* = 9–14.

We analyzed data with a two-way ANOVA using the strain and the diet as variation factors. For the percentage of time in open arms, the diet (*F*_(2, 69)_ = 4.192, *p* = 0.0191) and the interaction of diet and strain (*F*_(2, 69)_ = 4.607, *p* = 0.0132) were sources of variation; similar case for the percentage of entries to open arms (Diet, *F*_(2, 69)_ = 6.376, *p* = 0.0029; Interaction, *F*_(2, 69)_ = 7.185, *p* = 0.0015). The Tukey pairwise comparisons between diets revealed that only the WKY sucrose and shortening binge-type eaters exhibited increased anxiety-like behavior, showing a significantly lower percentage of time in open arms (control vs. sucrose *p* = 0.0399, vs. shortening *p* = 0.0003) and a significantly lower percentage of entries to open arms (control vs. sucrose *p* = 0.014, vs. shortening *p* < 0.0001) than their control group (Figure 6).

#### 3.3.2 Active coping behavior in the modified marble burying test

In addition to the EPM to evaluate the anxiety-type behavior, we measure the performance of coping behaviors as a response to the exposure to a novel situation, in this case, to a marble burying test arena. We analyzed data with a two-way ANOVA using the strain and the diet as variation factors for each behavior. For burying time, both factors and their interaction were sources of variation (Strain *F*_(1, 49)_ = 9.530, *p* = 0.0033; Diet *F*_(2, 49)_ = 3.556, *p* = 0.0361; Interaction *F*_(2, 49)_ = 8.123, *p* = 0.0009). In the case of compulsive rostral-grooming time, only the factors evaluated, not the interaction between them, were the source of variation (Strain *F*_(1, 49)_ = 16.07, *p* = 0.0002; Diet *F*_(2, 49)_ = 9.451, *p* = 0.0003).

**Figure 7:**
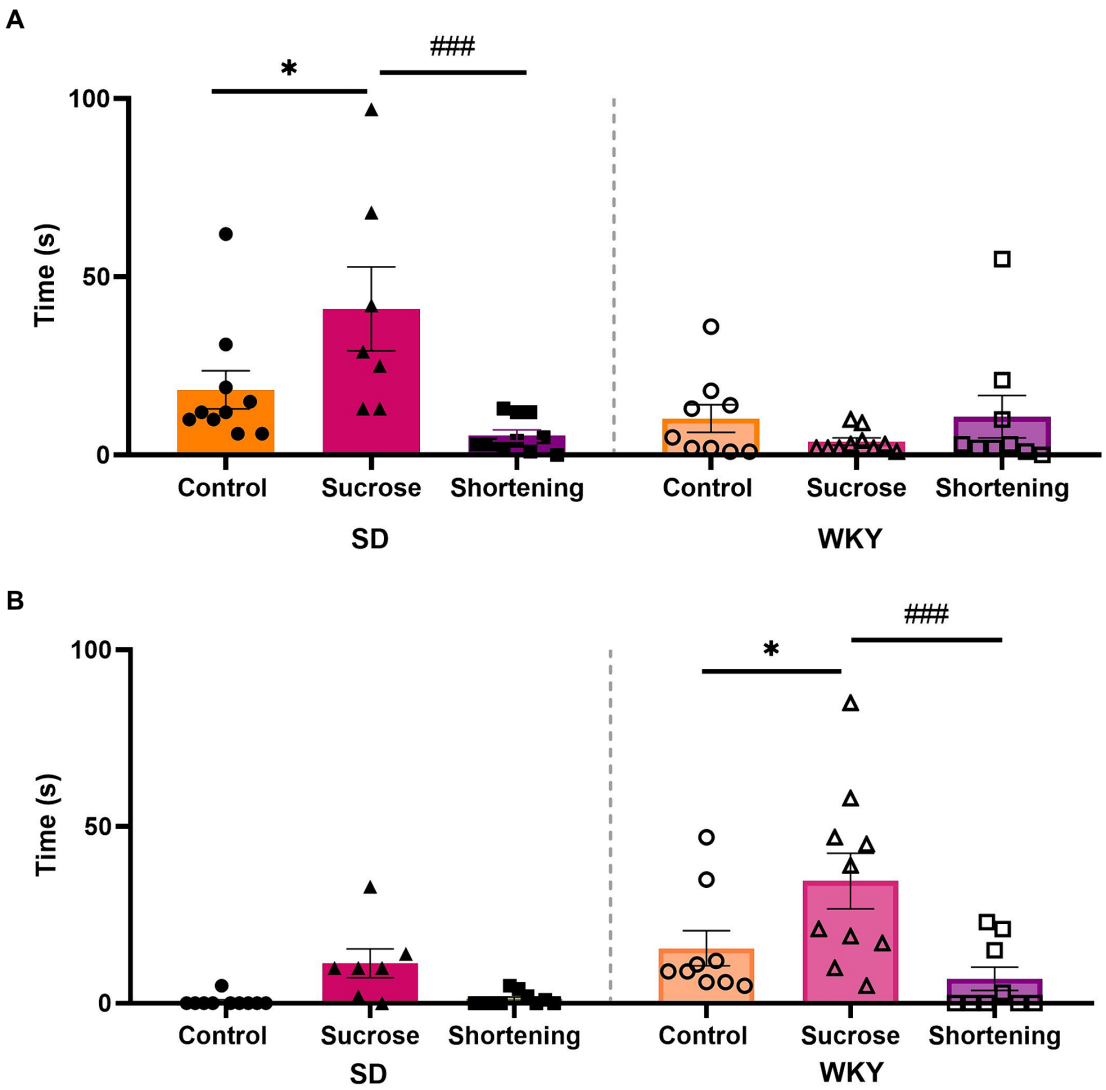
Burying **(A)** and compulsive rostral-grooming **(B)** time in the modified marble burying test of control, sucrose, and shortening groups of Sprague-Dawley (SD) and Wistar-Kyoto (WKY) rats subjected to the binge-type eating behavior induction protocol. Mean ± SEM. Two-way ANOVA. *Post-hoc*: Tukey * vs. Control, *p < 0.05; ^#^ vs. Sucrose, ^###^*p* < 0.001. *n* = 7–10.

Both strains differed in the predominant active coping behavior performed; while SD spent more time burying, WKY spent more time in compulsive rostral grooming. In both strains, the effect of diets on active coping behavior was the same; the Tukey pairwise comparisons between groups revealed that in both strains, the sucrose binge-type eaters exhibited increased anxiety-like behavior by showing a significantly higher time performing an active coping behavior (Figure 7).

### 3.4 Corticosterone response to stress

We evaluated the stress corticosterone response as an approximation to assess the effect of binge-type eating behavior on the activity of the HPA axis.

We performed a two-way ANOVA for each experimental group (control, sucrose, and shortening binge-type eaters), using the strain and the stress condition as variation factors. In general, stress exposure in WKY raised serum corticosterone levels, except in the sucrose binge-type eaters, who, basally, presented high corticosterone levels in a non-stress condition. For the SD strain, only the sucrose binge-type eaters were susceptible to the effect of stress exposure on the serum corticosterone level.

**Figure 8:**
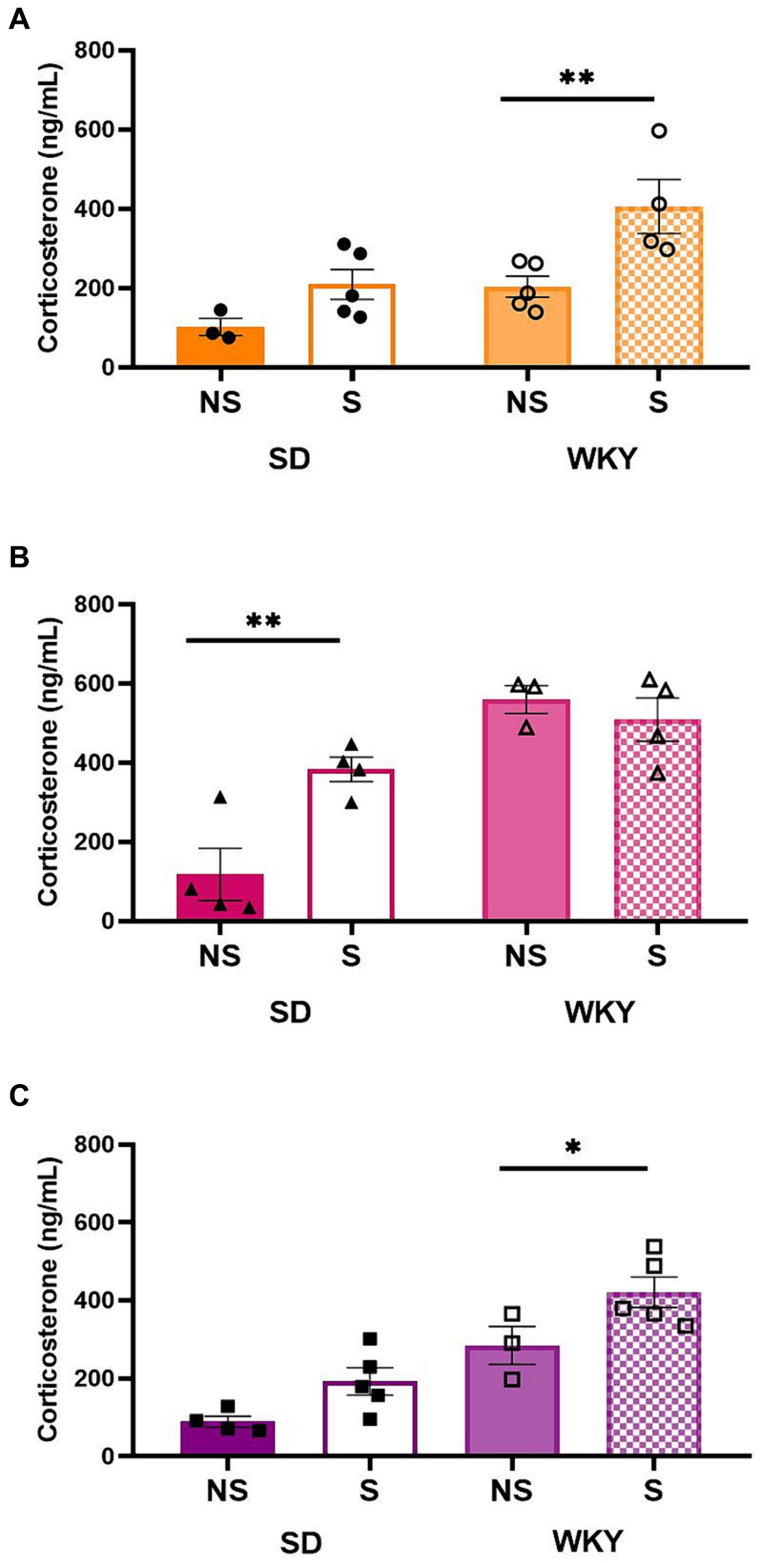
Corticosterone serum levels in control (chow) **(A)**, sucrose **(B)**, and shortening **(C)** groups of Sprague-Dawley (SD) and Wistar-Kyoto (WKY) rats in non-stressful (NS) and stressful (S) conditions. Mean ± SEM. Two-way ANOVA (analysis available on Supplementary material). *Post-hoc*: Sidak, *p < 0.05, **p < 0.01; *n* = 3–5.

For the control group and shortening binge-type eaters, both conditions were sources of variation; Sidack’s multiple comparisons between stress conditions revealed only in WKY a significantly higher serum corticosterone level in the stress condition than in its non-stress counterpart (Control *p* = 0.0091, Shortening *p* = 0.05). For sucrose binge-type eaters, the stress condition and the interaction between factors were the sources of variation; Sidack’s multiple comparisons revealed that only SD presented a significantly higher corticosterone level in its stress condition than in its non-stress counterpart (*p* = 0.0058) in the case of WKY, the corticosterone levels were increased in both stress conditions (Figure 8).

## 4 Discussion

Our results confirm the hypothesis that the high-stress sensitive phenotype is a common vulnerability factor for the development of binge-type eating and anxiety-like behavior since the WKY strain successfully showed both behaviors independent of the palatable food employed.

When comparing the performance of the WKY rat strain against the SD, the most used strain in binge-type eating studies (14), we demonstrated a significant difference between strains in response to an intermittent access model to palatable food despite successfully inducing high kilocalorie intake in both strains. The first differences observed during the development of the binge-type eating induction protocol were the different sizes between strains, the higher shortening consumption of WKY in comparison to SD, and the higher dispersion in the kilocalorie consumption between SD subjects in its three experimental groups in comparison with WKY.

Based on the different dispersion of the average kilocalorie intake between strains and the high difference in calorie density between the palatable foods employed (sucrose 30% solution: 1.3 kcal/g vs. shortening: 9 kcal/g), it was necessary to implement a classification criterion that helped us to identify those subjects that expressed an abnormally high kilocalorie intake. This way, we determine how much a “normal” kilocalorie intake would be. Since the high kilocalorie intake in our protocol was induced by intermittent palatable food access, we consider as “normal” consumption the kilocalorie intake expressed by rats with continuous palatable food access. Following this idea, we evaluated the kilocalorie intake in isolation sessions (like the isolation sessions of the binge-type eating induction protocol) of groups with sucrose or shortening *ad libitum*.

With this classification criterion, we identified SD rats resistant to developing binge-type eating behavior when using sucrose or shortening as palatable food and for WKY only when using sucrose, as all WKY with intermittent access to shortening were classified as binge-type eaters. Additionally, WKY required less palatable food access sessions than SD to develop abnormal eating behavior since a higher percentage of WKY sucrose and shortening binge-type eaters reached the classification criteria in only 2 weeks of access sessions. Based on the higher proportion of shortening binge-type eaters and the faster development of the binge-type eating behavior, we suggest that WKY is more susceptible to developing binge-type eating behavior when using shortening than SD.

Regarding the analysis and characterization of a binge episode, both strains presented equal kilocalorie consumption and consumption pattern in the sucrose groups, with nearly 60% of their consumption time taking place during the first 15 min; a similar pattern was observed in the WKY shortening group, but with a kilocalorie consumption significantly higher than SD in the same amount of time. For the SD shortening binge eaters, the consumption time was mainly distributed in two events with 45 min in between, differing from binge episode characteristics. Therefore, WKY but not SD show binge-eating episodes with both types of diet.

After analyzing the results, we determined that a rat’s binge episode lasts approximately 15 min. According to clinical studies, a human binge episode lasts approximately 2 h, the same duration as the isolation session used in the present study. However, as rats are significantly smaller than humans, their binge episodes are expected to be shorter. Therefore, we were interested in characterizing the duration and calorie consumption in binge episodes in rats. Nevertheless, it would be helpful in future research to track calorie consumption across the isolation session, measure the consumption rate, and confirm the occurrence of a binge eating episode since our consumption measures were performed at the end of the 2-h isolation session.

We were interested in evaluating if the main macronutrients of the palatable food (carbohydrates and lipids) could differentially affect anxiety and binge-type eating behavior development in WKY. We selected the sucrose 30% solution based on prior research with WKY (18) and shortening since it is one of the most used fats in SD binge-type eating models (29, 30). Although we employed two palatable foods with different macronutrient compositions, we did not compare their consumption due to the significant difference in kilocalorie density. However, in both cases, we confirm that regardless of the macronutrient composition of the palatable food, WKY is susceptible to developing a binge-type eating behavior by expressing a high kilocalorie intake in a short eating episode. This suggests that incorporating more complex and appealing foods that resemble those consumed by individuals with BED during their binge episodes may induce similar eating patterns in WKY. Furthermore, considering the potentiation effect of the carbohydrate-fat mixture on food reward (31), it is possible that such foods could facilitate the development of WKY binge-type eating behavior and express a higher consumption than SD, as happened with shortening.

In addition to the evaluation of binge-type eating susceptibility, we were interested in evaluating if our experimental animals simultaneously developed an anxiety-like behavior in response to intermittent access to sucrose or shortening, similar to what is observed in BED. In line with our prior research (18), intermittent access to sucrose led to anxiety-like behavior in WKY rats, as evidenced by results from the EPM test and the modified marble burying test. However, in SD rats, the induction of the anxiety-like behavior was only observed in the modified marble-burying test. The intermittent access to shortening also induced anxiety-like behavior in WKY only in the EPM with no effect on the SD strain.

In the modified marble-burying test, the effect of the diet on the anxiety-like behavior was the same between strains. However, the active coping behaviors performed were different. While SD spent more time burying, WKY spent more time compulsively rostral grooming. As previously described, the prevalence of compulsive rostral grooming instead of burying behavior as active coping behavior to stressor situations in WKY is a characteristic of this strain (32).

The expression of anxiety-like behavior in SD is something new, as it has been described in other protocols that it does not express any anxiety-like behavior associated with binge eating (15, 16). However, the anxiety evaluation in those protocols was performed by implementing the EPM test, a test where we also observed the absence of anxiety-like behavior. Taking into consideration the results of both applied anxiety tests, WKY is more susceptible than SD to developing anxiety-like behavior simultaneously with binge-type eating.

In evaluating serum corticosterone levels in response to a stress situation, we observed a differential effect of stress exposure on both strains. In SD control and shortening groups, the serum corticosterone levels did not increase in response to stress. It is possible that the lack of effect could be due to the nature of the stress used. It has been described that animals subjected to similar conditions to our stress sessions show a minimum rise in serum corticosterone due to the possibility of performing a cooping behavior during the event (28, 33). However, in WKY control and shortening groups, the stressor raised the corticosterone levels; this may be due to the hyperreactivity of the HPA axis, a trait of this strain (19). Additionally, binge sucrose sensitized the HPA axis in both strains since it facilitated the elevation of corticosterone levels in SD in response to a stressor that lacked an effect on its control group. In WKY, sucrose elevated the corticosterone levels even without stress, while no further rise was observed after stress exposure. This result can be interpreted as a blunted response, probably due to chronic over-activation.

The effect of binge sucrose on serum corticosterone levels matches the results obtained in the modified marble test; this reinforces the idea that binge sucrose intake alters the performance of the HPA axis. Some studies have described a blunted HPA axis response as one effect of the sucrose binge model in SD rats. However, the binge induction protocol used in those works differs from ours, particularly in implementing other stressors as a binge-inducing factor (34, 35) and the individual housing, which also affects the HPA axis response in binge-type eating models (36). In this way, the blunt response of the SD HPA axis in those binge-type eating models results from the binge sucrose plus the stressor exposure and the permanent isolation. In contrast, the HPA axis blunt response observed in WKY results from only the sucrose binge-type eating.

Present data suggest that the WKY strain represents an animal model adequate to resemble some traits of binge-eating disorder since it showed binge behavior with two types of high caloric content (sugar and shortening), developed the behavior in less time, and showed anxiety-like behavior. Furthermore, corticosterone response suggests alterations of HPA axis functions as it occurs in BED.

It is important to note that the current description of the SD and WKY strains as binge-type eating models was performed only on female rats. Although male rats have been described to exhibit higher levels of anxiety than females (37, 38), they have also been shown to consume less palatable food in binge-type eating protocols compared to females (39). In a previous study, we reported that male WKY rats exhibit lower consumption of sucrose in a binge-type eating model compared to females despite expressing a higher level of anxiety associated with sucrose binge eating (18). Based on the results exposed here, it would be interesting to evaluate if the anxiety-like behavior of male WKY is also induced by shortening binge-type eating or if it is only an effect of sucrose consumption. Additionally, we considered that comparing the amount of palatable food consumed between sexes, as reported (18, 39), is a limited perspective to compare the binge-type eating susceptibility between sexes. Therefore, implementing classification criteria based on “normal” palatable food consumption in males could better compare binge-type eating behavior between sexes.

One of the main limitations of this study, as we employed female rats, is the possible effect of the estrogen variation across the estrous cycle on the parameters evaluated here. Even though it has been previously reported that the estrous cycle did not affect palatable food consumption in some binge-type eating induction protocols, it has only been reported for the SD strain (34, 40), leaving the effect on the WKY strain unknown. More studies are needed to evaluate the possible effect of the estrous cycle on the female WKY binge-type eating and associated anxiety-like behavior.

In conclusion, the intermittence of palatable food alone induces binge-type eating behavior and anxiety-like behavior in rats with hyper-reactivity to stress without the implementation of any stressful stimulus or calorie restriction (41). In this way, female WKY offers a good model to study the relationship between anxiety-like and binge-type eating behaviors. However, as with other psychiatric animal models, there are some limitations based on the translatability of human-animal behavior that led to setting aside some disorder characteristic symptoms or involved social factors that are impossible to evaluate or emulate in the animal model. For example, in BED, the physical and mood discomfort after the binge episodes, the shame feeling associated with the binge episodes, and the absence of hunger when binging, among others. Nevertheless, the development of animal models provides the opportunity to identify novel hypotheses of neurobiological mechanisms that underlie the etiology of some abnormal behaviors associated with the psychiatric condition. To amplify the appearance validity of the WKY-binge eating model, we consider it important to evaluate the development of anxiety-like behavior when using a more complex palatable food since, based on our results, the binge of carbohydrates and fat affects the HPA axis function differently.

## Data availability statement

The raw data supporting the conclusions of this article will be made available by the authors, without undue reservation.

## Ethics statement

The animal study was approved by Comité Interno para el Cuidado y Uso de Animales de Laboratotio (CICUAL). The study was conducted in accordance with the local legislation and institutional requirements.

## Author contributions

DR-R: Conceptualization, Formal analysis, Investigation, Methodology, Writing – original draft. EE-C: Conceptualization, Formal analysis, Supervision, Writing – review & editing. CL-R: Conceptualization, Formal analysis, Investigation, Project administration, Resources, Supervision, Writing – review & editing.
