# Supplementary material for: Stress hyper-reactivity increases vulnerability to developing binge-type eating and associated anxiety-like behavior; comparison between Wistar-Kyoto and Sprague-Dawley rats
Source: Front Nutr. 2024 Apr 4;11:1368111. doi: 10.3389/fnut.2024.1368111 (PMC11024955; doi:10.3389/fnut.2024.1368111)
Supplement: Supplementary file 1 [file Table_1.docx]

| Supplementary Table 1. Average kilocalorie intake during the six 2 h isolation sessions of Sprague Dawley (SD) and Wistar Kyoto (WKY) groups with continuum palatable food access. Classification criteria to determine binge-type eating consumption. | | |
| --- | --- | --- |
| **Strain** | **Group** | **Consumption**  **(kcal/g body weight)** |
| SD | *Sucrose* | 0.1045 ± 0.003 |
|  | *Shortening* | 0.0899 ± 0.007 |
| WKY | *Sucrose* | 0.0888 ± 0.005 |
|  | *Shortening* | 0.0989 ± 0.008 |
| *Mean ± SED, n= 6.* | | |

Supplementary Material

| Supplementary Table 2. RM Two-way ANOVA of the body weight variations across the 12 isolation sessions of the binge-type eating induction protocol of the control, sucrose, and shortening groups of Sprague Dawley (SD) and Wistar Kyoto (WKY) rats, considering the isolation session and the strain as variation factors. | | | |
| --- | --- | --- | --- |
| **Two-way RM**  **ANOVA** | **Control** | **Sucrose** | **Shortening** |
| *Strain* | F _(1, 66)_ = 13.65 P=0.0005*** | F _(1, 90)_ = 8.549  P=0.0044*** | F _(1, 91)_ = 15.25  P=0.0002*** |
| *Isolation session* | F _(3.455, 228.0)_ = 161.0  P<0.0001*** | F _(2.596, 233.7)_ = 292.5  P<0.0001*** | F _(1.924, 175.1)_ = 234.9  P<0.0001*** |
| *Interaction* | F _(11, 726)_ = 9.739  P<0.0001*** | F _(11, 990)_ = 7.535  P<0.0001*** | F _(11, 1001)_ = 14.91  P<0.0001*** |

| Supplementary Table 3. Two-way ANOVA of the corticosterone serum levels in non-stressful and stressful conditions of the control, sucrose, and shortening groups of Sprague Dawley (SD) and Wistar Kyoto (WKY) rats, considering the stress condition and the strain as variation factors. | | | |
| --- | --- | --- | --- |
| **Two-way**  **ANOVA** | **Control** | **Sucrose** | **Shortening** |
| *Stress* | F _(1, 13)_ = 12.55  P=0.0036** | F _(1, 11)_ = 30.58  P=0.0002*** | F _(1, 13)_ = 10.67  P=0.0061** |
| *Strain* | F _(1, 13)_ = 11.64  P=0.0046** | F _(1, 11)_ = 4.388  P=0.0601 | F _(1, 13)_ = 33.41  P<0.0001*** |
| *Interaction* | F _(1, 13)_ = 1.179  P=0.2973 | F _(1, 11)_ = 9.467  P=0.0105* | F _(1, 13)_ = 0.2068  P=0.6568 |
